# Supplementary material for: Spatial signatures for predicting immunotherapy outcomes using multi-omics in non-small cell lung cancer
Source: Nat Genet. 2025 Oct 10;57(10):2482–93. doi: 10.1038/s41588-025-02351-7 (PMC12513832; doi:10.1038/s41588-025-02351-7)
Supplement: Supplementary file 1 — Supplementary Figs. 1–6. [file 41588_2025_2351_MOESM1_ESM.pdf]

nature genetics

Supplementary information https://doi.org/10.1038/s41588-025-02351-7

Spatial signatures for predicting immunotherapy outcomes using multi-omics in non-small cell lung cancer

In the format provided by the authors and unedited

Supplementary Information

Table of Contents

Supplementary Figure 1: The proportions of different cell types within tumor and stroma compartments in Yale cohort at PFS-5 years 2

Supplementary Figure 2: Univariable Analysis of Survival Based on Cell-types in the UQ Validation Cohort 3

Supplementary Figure 3: Generation and testing of resistance signatures in the stroma compartment and response signatures in tumor compartment 3

Supplementary Figure 4: Testing of resistance cell type signature in the tumor compartment of both Yale and UQ cohorts to predict OS-5-year and OS-2-year outcomes 4

Supplementary Figure 5: Testing of response cell type signature in the tumor compartment to predict overall survival 5

Supplementary Figure 6: Differences in raw unfiltered gene expression patterns between operators MM and TA 6

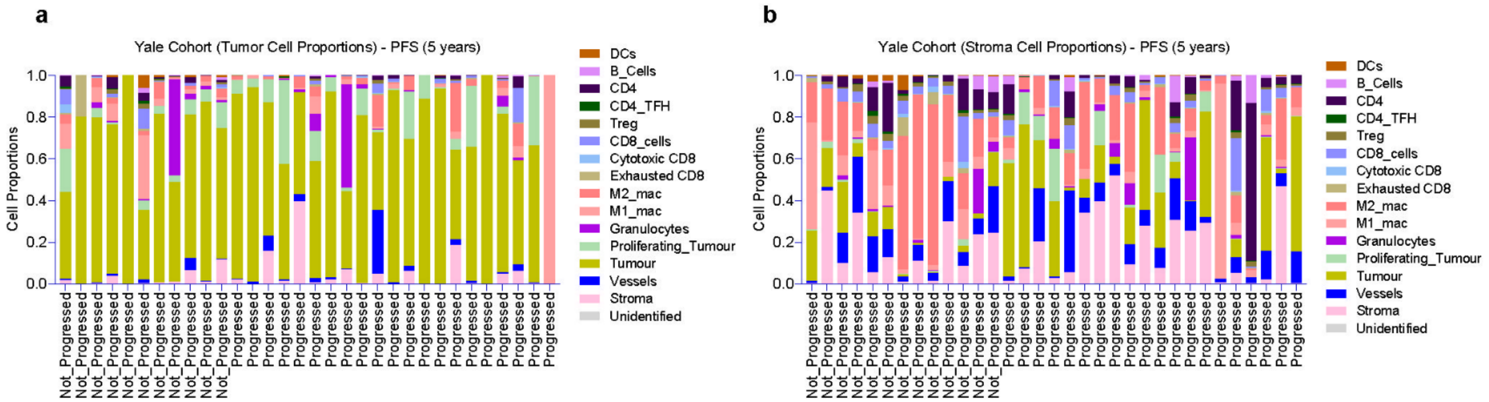

Supplementary Figure 1: The proportions of different cell types within tumor and stroma compartments in Yale cohort at PFS-5 years. (**a**) The proportions of different cell-types within the tumor compartment of the Yale cohort, categorized based on the progression free survival (PFS) 5-year index, illustrating the potential relationship between cellular composition and patient prognosis, is shown. (**b**) The distribution of different cell-types within the stroma compartment of the Yale cohort, categorized by the 5-year PFS index is shown.

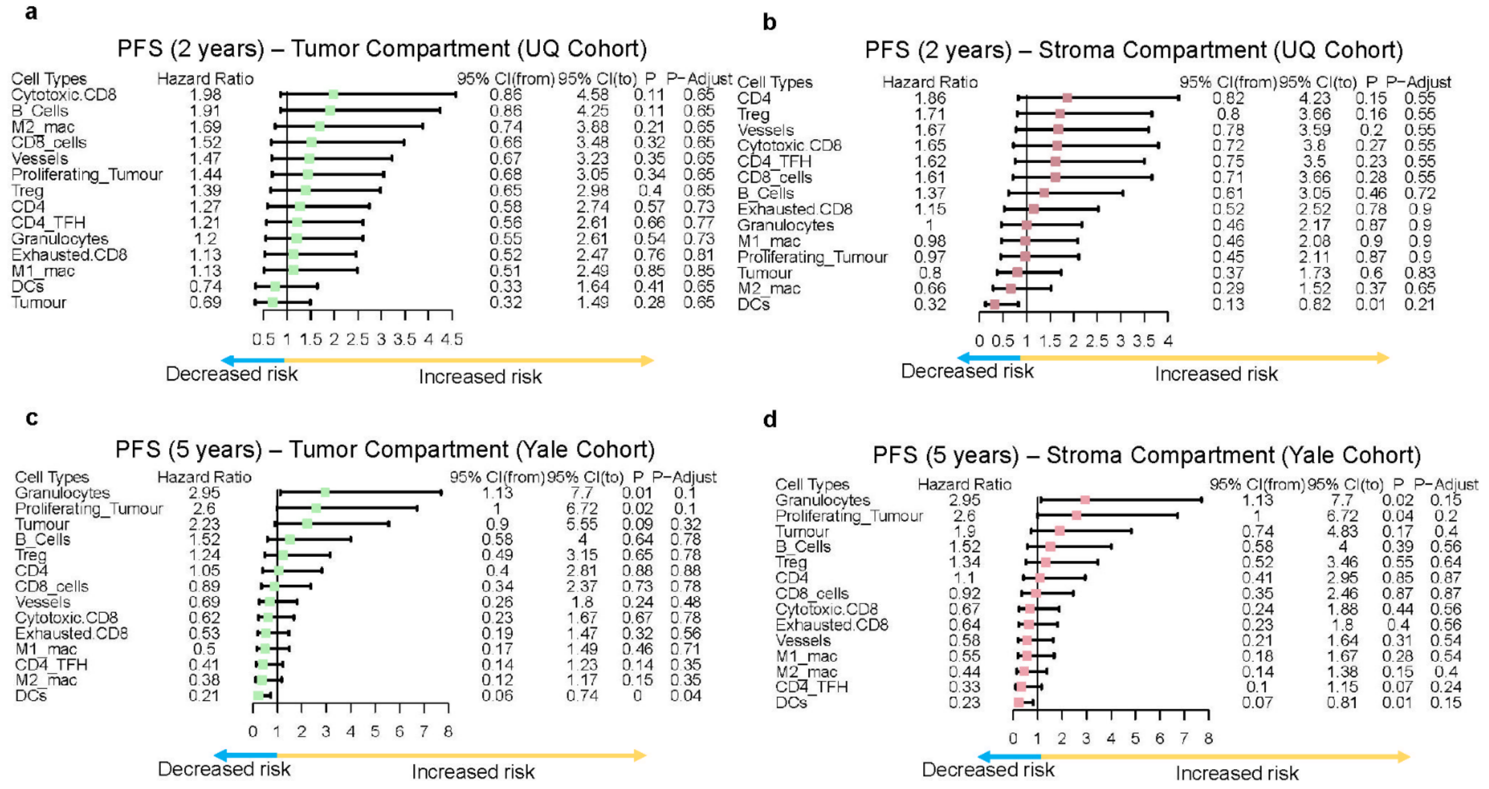

Supplementary Figure 2: Univariable Analysis of Survival Based on Cell-types in the UQ Validation Cohort. The PFS at 5 years in the tumor compartment (**a**) and the stromal compartment (**b**) of the Yale cohort, highlighting the impact of different cell types on the progression of the disease over the 5-year time frame, are shown. The tertile was used as a cut-point. The PFS at 5 years in the tumor compartment (**c)** and the stromal compartment **(d)** of the UQ cohort, highlighting the impact of different cell-types on the progression of the disease over the 5-year time frame. The tertile was used as a cut-point, as established in the Yale cohort.

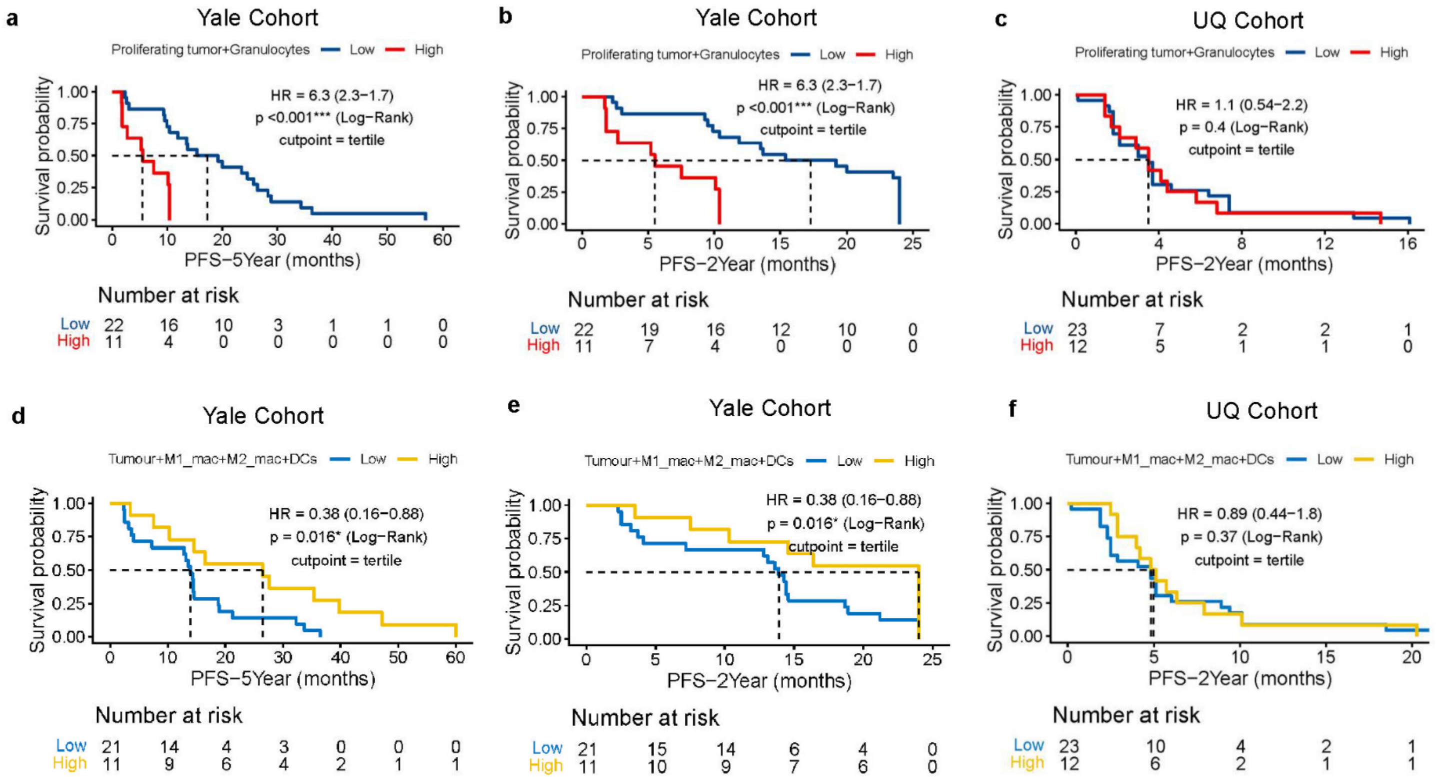

Supplementary Figure 3: Generation and testing of resistance signatures in the stroma compartment and response signatures in tumor compartment. (a) The resistant cell-type signature was trained in the stroma compartment to predict 5-year PFS. The KM plot demonstrates the performance metrics of the signature during training. (**b**) The trained resistance cell type signature was tested in the stroma compartment of the Yale cohort to predict 2-year PFS. The KM survival curve shows the predictive capability of the signature for short-term survival in the Yale cohort. (**c)** The resistance cell-type signature was validated in the stroma compartment of the UQ cohort to predict 2-year PFS. The KM plot shows the performance of the signature. (**d**) The response signature was trained in the tumor compartment of the Yale cohort to predict 5-year PFS. The KM plot shows the associated performance metrics. **(e)** The response signature was tested in the

tumor compartment of the Yale cohort to predict 2-year PFS. The KM survival curve illustrates predictive performance. (**f**) The response signature was validated in the tumor compartment of the UQ cohort to predict 2-year PFS. The KM survival curve illustrates the performance metrics to confirm the signature's predictive validity. 2-tailed and 1-tailed Log-Rank tests are used on the discovery and validation cohorts respectively, where the direction of the effect in the latter is chosen to match the direction of the effect in the former.

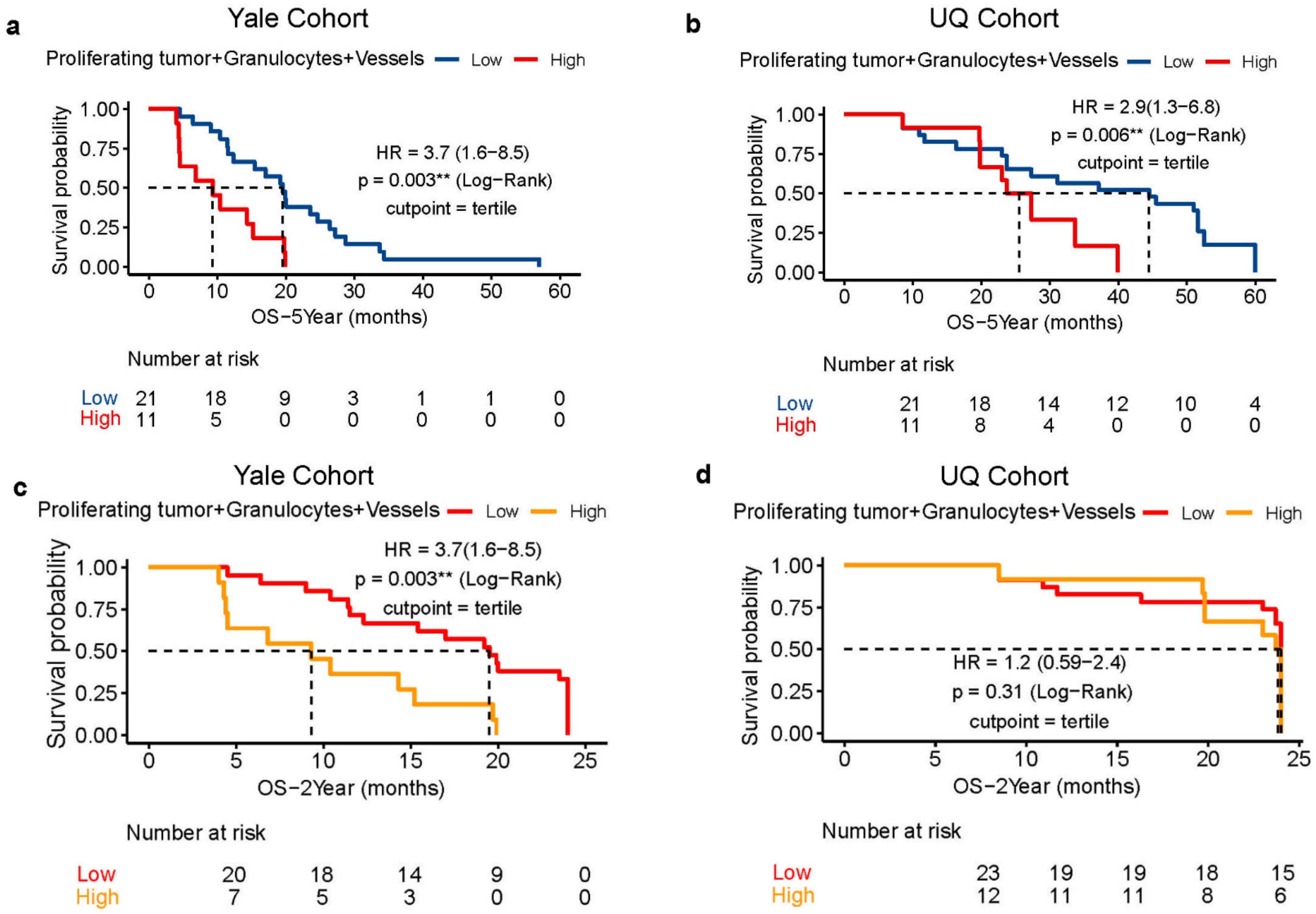

Supplementary Figure 4: Testing of resistance cell type signature in the tumor compartment of both Yale and UQ cohorts to predict OS-5-year and OS-2-year outcomes. (a) The resistance cell type signature was tested in the tumor compartment of the Yale cohort to predict 5-year overall survival (OS-5 years). The Kaplan-Meier survival curve shows the stratification of patients based on the presence of the resistance cell type signature, highlighting its prognostic value. (**b)** The resistance cell type signature was evaluated in the tumor compartment of the UQ cohort to predict 5-year overall survival (OS-5 years). The KM survival curve demonstrates the ability of the signature to distinguish between patients

with different survival outcomes, confirming its predictive utility. (**c**) The resistance cell type signature was assessed in the tumor compartment of the Yale cohort to predict 2-year overall survival (OS-2 years). The KM survival curve illustrates the prognostic significance of the signature, showing its impact on short-term survival predictions. (**d**) The resistance cell type signature was tested in the tumor compartment of the UQ cohort to predict 2-year overall survival (OS-2 years). The KM survival curve reveals the signature's effectiveness in predicting short-term survival outcomes. 2-tailed and 1-tailed Log-Rank tests are used on the discovery and validation cohorts respectively, where the direction of the effect in the latter is chosen to match the direction of the effect in the former.

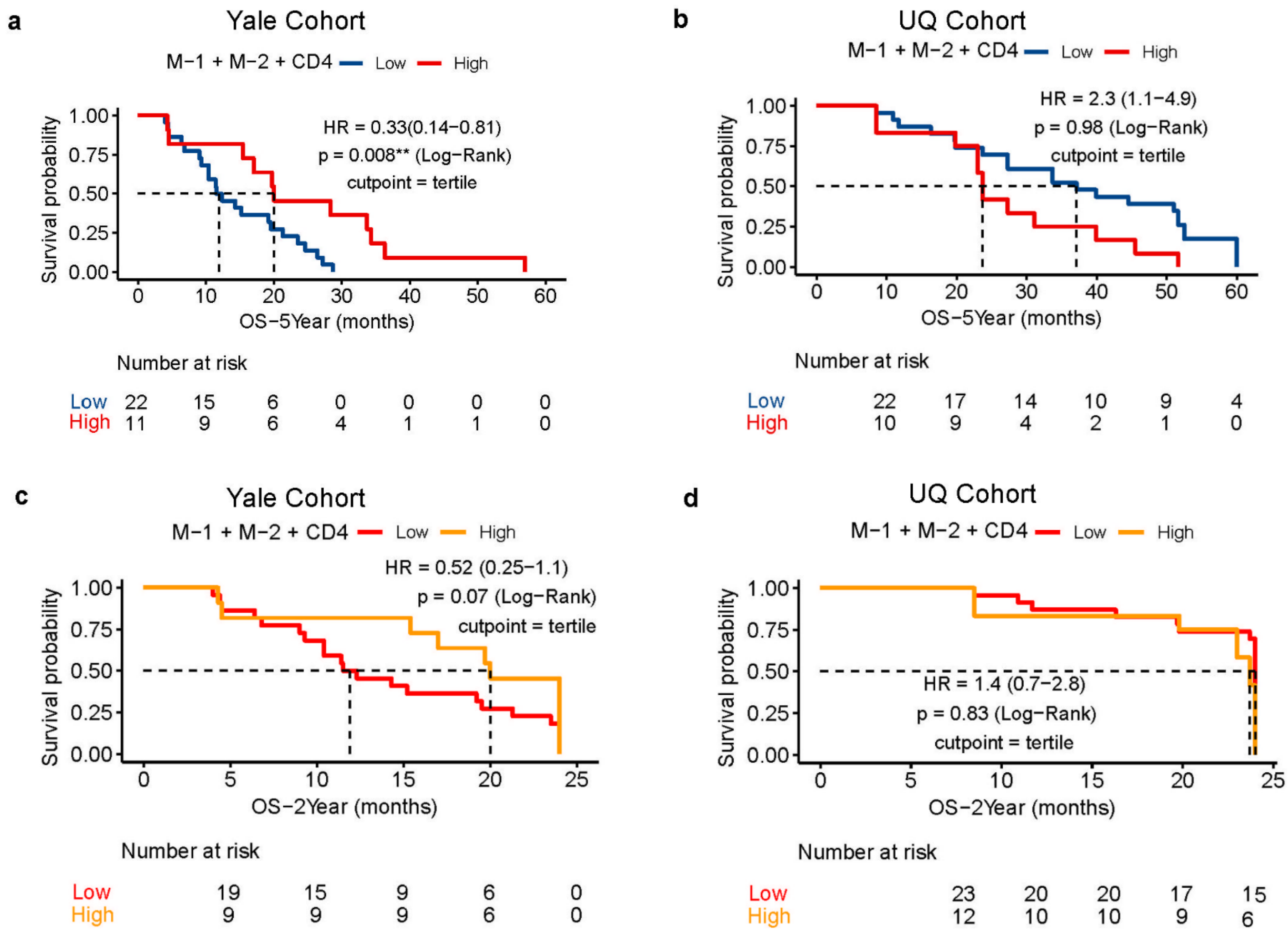

Supplementary Figure 5: Testing of response cell type signature in the tumor compartment to predict overall survival. (a) The response cell type signature was tested in the tumor compartment of the Yale cohort to predict 5-year overall survival (OS-5 years). The KM survival curve displays patient stratification based on the presence of the response cell type signature, highlighting its prognostic value for long-term survival. (**b**) The response cell type signature was

evaluated in the tumor compartment of the UQ cohort to predict 5-year overall survival (OS-5 years). The KM survival curve demonstrates the differentiation between patients with varying survival outcomes, confirming its predictive utility for long-term prognosis. **(c)** The response cell type signature was assessed in the tumor compartment of the Yale cohort to predict OS 2-year. The KM survival curve illustrates the prognostic significance of the signature, showing its impact on 2-year survival predictions. **(d)** The response cell type signature was tested in the tumor compartment of the UQ cohort to predict OS 2-year. 2-tailed and 1-tailed Log-Rank tests are used on the discovery and validation cohorts respectively, where the direction of the effect in the latter is chosen to match the direction of the effect in the former.

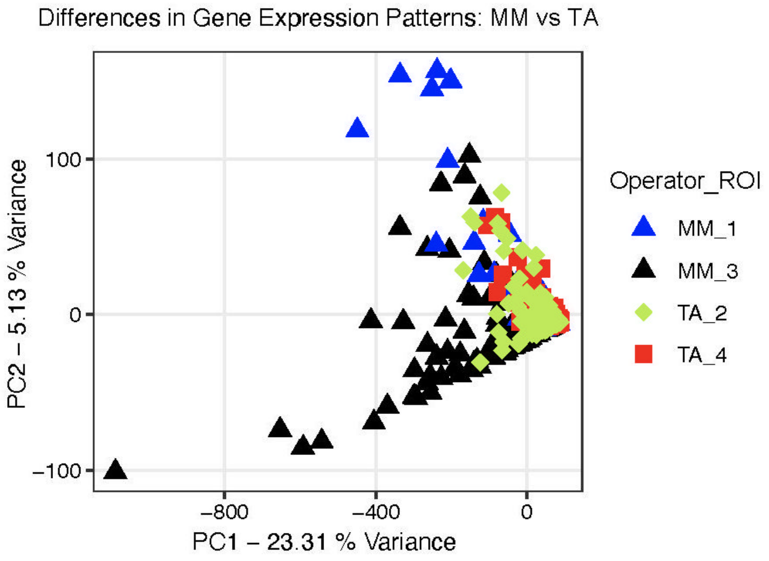

Supplementary Figure 6: Differences in raw unfiltered gene expression patterns between operators MM and TA. Principal component analysis (PCA) illustrates the variance in percentages among the experimental replicates. MM conducted experiments 1 and 3, while TA conducted experiments 2 and 4. The variance between MM_1, TA_2, and TA_4 was minimal, whereas the variance between MM_3 and the rest was 23.31%.
