# Supplementary material for: Spatial signatures for predicting immunotherapy outcomes using multi-omics in non-small cell lung cancer
Source: Nat Genet. 2025 Oct 10;57(10):2482–93. doi: 10.1038/s41588-025-02351-7 (PMC12513832; doi:10.1038/s41588-025-02351-7)
Supplement: Supplementary file 2 — Reporting Summary [file 41588_2025_2351_MOESM2_ESM.pdf]

nature portfolio

Corresponding author(s): David L Rimm, Arutha Kulasinghe

Last updated by author(s): Jul 17, 2025

Reporting Summary

Nature Portfolio wishes to improve the reproducibility of the work that we publish. This form provides structure for consistency and transparency in reporting. For further information on Nature Portfolio policies, see our Editorial Policies and the Editorial Policy Checklist.

Statistics

For all statistical analyses, confirm that the following items are present in the figure legend, table legend, main text, or Methods section.

n/a	Confirmed	
☐	☒	The exact sample size (n) for each experimental group/condition, given as a discrete number and unit of measurement
☐	☒	A statement on whether measurements were taken from distinct samples or whether the same sample was measured repeatedly
☐	☒	The statistical test(s) used AND whether they are one- or two-sided<br>*Only common tests should be described solely by name; describe more complex techniques in the Methods section.*
☐	☒	A description of all covariates tested
☐	☒	A description of any assumptions or corrections, such as tests of normality and adjustment for multiple comparisons
☐	☒	A full description of the statistical parameters including central tendency (e.g. means) or other basic estimates (e.g. regression coefficient) AND variation (e.g. standard deviation) or associated estimates of uncertainty (e.g. confidence intervals)
☐	☒	For null hypothesis testing, the test statistic (e.g. F, t, r) with confidence intervals, effect sizes, degrees of freedom and P value noted<br>*Give P values as exact values whenever suitable.*
☒	☐	For Bayesian analysis, information on the choice of priors and Markov chain Monte Carlo settings
☐	☒	For hierarchical and complex designs, identification of the appropriate level for tests and full reporting of outcomes
☒	☐	Estimates of effect sizes (e.g. Cohen's d, Pearson's r), indicating how they were calculated

Our web collection on statistics for biologists contains articles on many of the points above.

Software and code

Policy information about availability of computer code

Data collection	Commercial Software<br>1) Akoya Biosciences PCF (Phenocycler Fusion) Platform: This platform's associated software was used for the analysis of spatial proteomic data, including image processing and data export in qptiff format for further analysis.<br>2) GeoMx DSP (Digital Spatial Profiling) Platform by Nanostring Technologies: The software provided by Nanostring was used for the analysis of whole transcriptome (WTA) and cancer transcriptome (CTA) data, including processing sequencing data and generating digital count conversion (.dcc) files.
Data analysis	Open-Source Software<br>1) QuPath (v0.4.2), 2) Cellpose (v2.0), 3) Scanpy (Version: 1.8.2), 4) Harmonypy (v 0.0.5), 5) Phenograph (v1.5.7), 6) CIBERSORTx, 7) Scimap, 8) R (v4.2.1), 9) mastR (v1.8.0), and 10) glmnet (v4.1-9).<br>Custom Scripts<br>Custom scripts were developed for data preprocessing, integration, clustering, and the development of cell type and gene signatures. These scripts include the processing pipelines for the Phenocycler Fusion data, as well as the Cox regression modeling with LASSO regularization. These scripts are available on GitHub at https://github.com/tznaung/NSCLC_SpatialOmics.

For manuscripts utilizing custom algorithms or software that are central to the research but not yet described in published literature, software must be made available to editors and reviewers. We strongly encourage code deposition in a community repository (e.g. GitHub). See the Nature Portfolio guidelines for submitting code & software for further information.

Data

Policy information about availability of data

All manuscripts must include a data availability statement. This statement should provide the following information, where applicable:

- Accession codes, unique identifiers, or web links for publicly available datasets
- A description of any restrictions on data availability
- For clinical datasets or third party data, please ensure that the statement adheres to our policy

Raw and processed DSP-GeoMx WTA RNA sequencing data from the Yale discovery cohort and the Greek validation cohort are available under accession number GSE271689. DSP-CTA raw RNA sequencing data of UQ validation cohort can be assessed via GSE221733. Further data requests should be directed to the corresponding authors.

Research involving human participants, their data, or biological material

Policy information about studies with human participants or human data. See also policy information about sex, gender (identity/presentation), and sexual orientation and race, ethnicity and racism.

Reporting on sex and gender

Sex-Based Analysis
The study did not specifically report sex-based analyses due to the primary focus on developing and validating cell type and gene signatures associated with immunotherapy outcomes. The datasets used for this analysis were primarily stratified by clinical outcomes (e.g., progression-free survival) rather than sex or gender. Therefore, the influence of sex or gender on these outcomes was not assessed in the primary analyses.
Justification for Lack of Sex- and Gender-Based Analysis
The absence of sex- and gender-based analyses in this study is due to the research design, which aimed to develop predictive models based on cell type and gene expression profiles across the entire patient population. Given the exploratory nature of this study and the focus on biomarker discovery, the analysis was not stratified by sex or gender. Future studies may consider sex- and gender-specific differences if they are deemed relevant to the specific biomarkers or treatment responses being investigated.

Reporting on race, ethnicity, or other socially relevant groupings

Race, ethnicity, and other socially relevant groupings were not explicitly analyzed in this study. To control for confounding variables, we focused on patients receiving PD-1 based immunotherapy as their first-line treatment, standardizing treatment exposure across cohorts. We also used machine learning models and Cox regression with LASSO regularization, which inherently controls for multiple variables simultaneously, reducing bias. Additionally, cell type and gene signature analyses were performed independently of demographic factors, aiming to identify robust biomarkers associated with treatment outcomes, regardless of race or ethnicity. Future studies may explore these factors where relevant.

Population characteristics

Population Characteristics

The study included three independent cohorts of NSCLC patients treated with PD-1 based immunotherapies.
Yale Cohort (Training Set)
Sample Size: 113 tissue samples, narrowed to 41 first-line only immunotherapy treated patients for final analysis
Age: Median age of participants was 69.
Diagnosis: Advanced or metastatic NSCLC.
Treatment: First-line PD-1 based immunotherapy.
Covariates: Included clinical characteristics such as age, sex, tumor stage, histology, prior chemotherapy, smoking status, type of immunotherapy and treatment history.

UQ Cohort (Validation Set)
Sample Size: 42 tissue samples, narrowed to patients for final analysis.
Age: Median age of participants was 63.
Diagnosis: Recurrent NSCLC post-surgery.
Treatment: First-line PD-1 based immunotherapy.

Greek Cohort Cohort (Training Set)
Sample Size: 79 tissue samples, narrowed to 61 first-line only immunotherapy treated patients for final analysis.
Age: Median age of participants was 70.
Diagnosis: Advanced or metastatic NSCLC.
Treatment: First-line PD-1 based immunotherapy.
Covariates: Included clinical characteristics such as age, sex, tumor stage, histology, prior chemotherapy, smoking status, type of immunotherapy and treatment history.

Recruitment

Tissue samples were retrospectively collected from Yale Cancer Center (YCC), the University of Queensland (UQ), and the University of Anthems (Greece). The Yale cohort included samples collected between 2012 and 2019, the UQ cohort between 2009 and 2018, and the Greek cohort between 2019 and 2023. Retrospective collection was employed to minimize prospective bias and ensure representative data aligned with the study objectives.

Ethics oversight

The study protocol was approved by the Yale Human Investigation Committee (Protocol #95050082199) for the Yale Cancer Center cohort; by the Queensland University of Technology Human Research Ethics Committee (Protocol #2000000494), ratified by the University of Queensland, for the UQ cohort; and by the Ethics Committee of Sotiria General Hospital, Medical School (HIC Protocol #16760/23-06-2023) for the Greek cohort.

Note that full information on the approval of the study protocol must also be provided in the manuscript.

Field-specific reporting

Please select the one below that is the best fit for your research. If you are not sure, read the appropriate sections before making your selection.

☒ Life sciences ☐ Behavioural & social sciences ☐ Ecological, evolutionary & environmental sciences

For a reference copy of the document with all sections, see nature.com/documents/nr-reporting-summary-flat.pdf

Life sciences study design

All studies must disclose on these points even when the disclosure is negative.

Sample size	No statistical method was used to predetermine sample size. Sample sizes were based on the availability of tissue samples collected during the specified periods: 2012–2019 for the Yale cohort, 2009–2018 for the University of Queensland cohort, and 2019–2023 for the Greek cohort. All available samples that met the inclusion criteria were included in the analysis to maximize the use of existing retrospective data and resources.
Data exclusions	One region of interest (ROI) was excluded from the analyses due to data inconsistencies observed between two operators (TNA and MM) during the analysis of gene expression patterns using the DSP-GeoMx-WTA platform. Although potential issues were identified prior to sequencing, the experiment was carried out to determine whether the data would remain usable. Results from this ROI differed significantly from the rest of the dataset and were deemed unreliable for inclusion. This exclusion was predetermined and is documented in Extended Data Figure. 8. The remaining data were not excluded and were used to develop gene signatures of resistance and response using a LASSO-based framework within the tumor compartment of advanced NSCLC.
Replication	To verify the reproducibility of the experimental findings from the Yale NSCLC cohort, we validated the LASSO-generated cell type models in an external cohort from the University of Queensland (UQ), Australia. All replication attempts were successful, confirming the generalizability of the identified cell-type signatures. For the gene signatures, validation was performed across two independent external cohorts from Australia and Europe. These replication efforts were also successful, supporting the robustness, reproducibility, and broader applicability of the identified gene models.
Randomization	In this study, participants were not prospectively randomized into experimental groups. Instead, during model development, samples from the Yale cohort were randomly partitioned into training and testing sets as part of a cross-validation strategy within the LASSO framework. This re-sampling approach helped to minimize bias and control for covariates during the development of resistance and response-specific cell type and gene signatures. Given the retrospective nature of the study, this method provided a rigorous and reproducible means of model evaluation.
Blinding	Blinding was not applicable to this study, as the focus was on analyzing pre-existing tissue samples and gene expression data using computational methods. Since the study involved retrospective data analysis rather than intervention-based experimentation, group allocation and investigator bias were minimized by the automated, objective nature of the LASSO framework.

Reporting for specific materials, systems and methods

We require information from authors about some types of materials, experimental systems and methods used in many studies. Here, indicate whether each material, system or method listed is relevant to your study. If you are not sure if a list item applies to your research, read the appropriate section before selecting a response.

Materials & experimental systems

n/a	Involved in the study	
☐	☒	Antibodies
☒	☐	Eukaryotic cell lines
☒	☐	Palaeontology and archaeology
☒	☐	Animals and other organisms
☐	☒	Clinical data
☒	☐	Dual use research of concern
☒	☐	Plants

Methods

n/a	Involved in the study	
☒	☐	ChIP-seq
☒	☐	Flow cytometry
☒	☐	MRI-based neuroimaging

Antibodies

Antibodies used	In this study, the following nanostring antibodies were used to define regions of interest (ROIs) by fluorescent masking.<br>1. CD45 (leukocyte marker): Supplier NanoString Technologies<br>2. PanCK (Tumor compartment marker):Supplier: NanoString Technologies<br>3. CD68 (Macrophage marker):Supplier: NanoString Technologies<br>The following Akoya antibodies were used for for cell phenotyping, functional subset identification by CODEX<br>4. CD31 (Endothelial cell marker):Supplier: Akoya Biosciences

5. CD4 (T helper cell marker):Supplier: Akoya Biosciences
6. HLA-A (human leukocyte antigen): Supplier: Akoya Biosciences
7. CD44 (cancer stem cells): Supplier: Akoya Biosciences
8. CD20 (B-cell marker):Supplier: Akoya Biosciences
9. SMA (Smooth Muscle Actin marker):Supplier: Akoya Biosciences
10. E-cadherin (Epithelial cell marker):Supplier: Akoya Biosciences
11. CD68 (Macrophage marker):Supplier: Santa Cruz; clone; KP1, catalog: sc-20060
12. CD45RO (memory T cells marker): Supplier: Akoya Biosciences
13. Pan-cytokeratin (epithelial marker):Supplier:Akoya Bioscience
14. CD45 (leukocyte marker): Supplier Akoya Biosicences
15. Vimentin (Mesenchymal cell marker):Supplier: Akoya Biosciences
16. CD11b (Myeloid cell marker):Supplier: Akoya Biosciences
17. CD11c (Dendritic cell marker):Supplier: Akoya Biosciences
18. CD34 (Endothelial progenitor cell marker):Supplier: Akoya Biosciences
19. CD8 (Cytotoxic T-cell marker):Supplier: Akoya Biosciences
20. IDO1(Tryptophan regulation):Supplier:Akoya Biosciences
21. FOXP3 (T regulatory cell marker):Supplier: Akoya Biosciences
22. CD21 (B cells and follicular dendritic cells marker): Supplier: Akoya Biosciences
23. CD14 (Monocyte/Macrophage marker):Supplier: Akoya Biosciences
24. Granzyme B (Cytotoxic T-cell marker):Supplier: Akoya Biosciences
25. Collagen IV (an antibody that binds to Type IV collagen): Supplier: Akoya Biosciences
26. PD-L1 (Tumor cell marker):Supplier: Akoya Biosciences
27. CD3e (T-cell marker):Supplier: Akoya Biosciences4
28. PD-1 (Exhausted T-cell marker):Supplier: Akoya Biosciences
29. Ki67 (Proliferation marker):Supplier: Akoya Biosciences
30. ICOS (T Follicular helper cell marker):Supplier: Akoya Biosciences
31. LAG3 (immune checkpoint): Supplier: Akoya Biosciences
32. CD163 (M2 Macrophage marker):Supplier: Akoya Biosciences

Validation

The validation of each primary antibody used in this study was conducted as follows:
Antibodies from Akoya Biosciences and NanoString Technologies: All antibodies obtained from Akoya Biosciences and NanoString Technologies were fully validated by the manufacturers for the species and applications used in this study. These antibodies are routinely employed in their respective platforms (Phenocycler Fusion and GeoMx DSP) and have been rigorously tested to ensure specificity and reliability. Validation statements provided by the manufacturers affirm their appropriateness for the species and experimental conditions applied in our research. Application note for antibody validation by nanostring can be found: https://nanostring.com/wp-content/uploads/WP_GeoMx_Antibody_Validation_White_Paper.pdf.
application note for antibody validation for akoya can be found https://www.akoyabio.com/wp-content/uploads/2022/01/Phenocycler_Technical-Note_Validation-of-Commercial_DN-00140.pdf aswell as in original CODEX Cell publication https://pubmed.ncbi.nlm.nih.gov/32763154/
CD68 (Clone KP1) from Santa Cruz Biotechnology: The CD68 antibody (Clone KP1) sourced from Santa Cruz Biotechnology has been validated by the manufacturer for human tissue and immunohistochemical applications. This antibody is widely recognized in the scientific community and has been cited in over 200 peer-reviewed publications, demonstrating its reliability and effectiveness in similar studies. The extensive use and validation of this clone across numerous studies further support its suitability for the applications in our research.

Clinical data

Policy information about clinical studies
All manuscripts should comply with the ICMJE guidelines for publication of clinical research and a completed CONSORT checklist must be included with all submissions.

Clinical trial registration

This study is retrospective and does not involve a clinical trial; therefore, it does not have a trial registration number.

Study protocol

As this study is retrospective and not a clinical trial, a formal trial protocol is not available.

Data collection

Data collection took place in three primary settings:

1. Yale Cancer Center (YCC), New Haven, CT, USA
Setting: A comprehensive cancer center within a large academic medical institution.
Time Period: Tissue samples were collected retrospectively between 2012 and 2019.

2. University of Queensland (UQ), Brisbane, Australia
Setting: A leading research university and affiliated hospitals specializing in oncology.
Time Period: Tissue samples were collected retrospectively between 2009 and 2018.

3. Sotiria General Hospital, Athens, Greece
Setting: A major academic hospital affiliated with the Medical School in Greece, specializing in pulmonary diseases and oncology.
Time Period: Tissue samples were collected retrospectively between 2019 and 2023.

Outcomes

Primary Outcome Measures
The primary outcome measure was the progression-free survival (PFS) at 2 years in patients with non-small cell lung cancer (NSCLC) treated with PD-1 based immunotherapies. PFS was defined as the time from the start of treatment to disease progression or death from any cause. This measure was assessed using clinical data and imaging studies in accordance with the Response Evaluation Criteria In Solid Tumors (RECIST) version 1.1.

Secondary Outcome Measures
The secondary outcome measures included the development and validation of cell type-specific and gene expression signatures predictive of treatment response or resistance. These measures were assessed using spatial proteomic and transcriptomic profiling data, processed and analyzed through a LASSO-based Cox regression framework. The performance of these signatures was evaluated by their ability to predict PFS in independent validation cohorts.
Both outcome measures were pre-defined based on the study's objectives to understand the biomarkers associated with immunotherapy outcomes in NSCLC.

Plants

Seed stocks	not applicable
Novel plant genotypes	not applicable
Authentication	not applicable
